# How It Is Done

**Published:** 1876-03

**Authors:** 


### *HOW IT IS DONE.*

When we send an old, soiled coat to the scourer to be cleaned, and get it back not only free from spots, but actually freshened up and improved in color, we wonder how it is done. The mechanic who does the work is very careful not to tell us, and no amount of experimenting on our part produces a kindred result. We may soap and brush and scrub and apply benzine and alcohol until we are tired out, and if we succeed in eradicating the spot we are almost sure to leave the garment looking worse than before. Our "grease extractor" leaves an ugly circle around the original spot, and we decide that we do not amount to much as a cleanser of soiled garments.

A week ago one of our leading merchant-tailors let us into the secret. It appears that there is a fluid, the ingredients of which are concealed, which the professional scourers use, and by the aid of which these beautiful results are secured. It is called "Ormond's Magic Cleanser." The manufacture of this fluid for "the trade"—that is, for clothes-cleaners and tailors—is a great business. No effort has been made to attract the attention of the public at large to its use.

We thought we had got a new idea, and, as usual, we concluded to follow it up. We asked the address of the maker, and learned that the proprietor of the cleanser was Mr. H. Wiswell, of No 55 Leonard Street. We called on him, and told him that we were interested to know more about a preparation which we had heard highly commended. He did not tell us how he made it, but he did show us the names of a multitude of his customers, here and elsewhere. We found on the list the leading tailors of New York, and scourers innumerable. Among them we recognized the names of Brooks Brothers, Matthew Rock, T. H. Miller, R. S. Mann, J. D. Powers, Abbott & Duyckirk, A. J. Dewey, Drifenthaler & Lander, J. Q. Laws, T. P. Saunders, Mortimer Brown, Leiss Brothers, N. E. Mead, Traphagen, Hunter & Co., Watkins & Lewis, P. C. Barnum & Co., Charles Emrich & Co., C. M. Church & Son, Gordon Brothers, and over 200 others in New York and Brooklyn.

The method of using the "Cleanser" was explained to us. For removing grease, paint, or spots of any kind from woolen, alpaca, or silk garments, felt hats, shawls, carpets, etc., or for cleaning collars of coats, the plan is to beat the dust well out and brush off before applying the liquid. Shake the bottle before using. For dark-colored goods use a woolen rag or sponge saturated with the liquid, and rub well in until the stain is removed; then rinse off with water and apply a damp rag and press off with a warm iron. For light colors and blue shades take one wine glassful to a pint of hot water. If very much soiled, use a nail brush and rinse off with water, and finish by pressing with a warm iron on a damp rag. For paint or tar, put a drop of oil on the spot to soften it before using the liquid. On all thin articles place a woolen rag under to absorb the liquid. To wash a whole dress, take half a pint of the liquid to a pail of hot water.

It is wonderful to observe how greatly the appearance of all fabrics is improved by this application. The color seems to be renewed and refreshed, and an air of newness is at once taken on. It is clear to our mind that the article should be in every house. Heretofore it has been sold by the gallon, at $2.50. In future, druggists will be able to order it in bottles, to be sold at 25 cents. We believe that wherever it gains a foothold there it will remain. Those wishing to test it can address Mr. Wiswell, as above.

---

Comfortable Inner Soles.—Mr. William C. Leibrandt, of Wilmington, Delaware, makes very nice inner-soles, which he calls "Foot-Warmers." We are indebted to this gentleman for some specimens, and have given them a trial. They are certainly very comfortable and useful affairs. Being made of felted horsehair, they are soft and yielding, and impart a gentle friction to the feet in the act of walking. We think them the best things we have yet seen for those whose feet perspire, and who accordingly suffer from damp and chilly feet. Our excellent friend, Mr. George G. Lobdell, of Wilmington, uses them constantly and commends them warmly.

---

Harpers' Magazine, for March, commences with a finely-illustrated article, entitled, "The Principalities of the Danube." With this number is concluded the interesting series of articles entitled, "The First Century of the Republic."

